# Promoting the Mediterranean Diet in the Workplace: An Observational Cross-Sectional Study

**DOI:** 10.3390/nu18193132

**Published:** 2026-09-23

**Authors:** Nicola Magnavita, Igor Meraglia, Giacomo Viti, Martina Gasbarri, Lucia Isolani

**Affiliations:** 1 Department of Safety and Bioethics, Università Cattolica del Sacro Cuore, Largo F. Vito 1, 00168 Roma, RM, Italy; meraglia.igor93@gmail.com (I.M.); giacomo.viti@aslroma4.it (G.V.); 2 Lavoro Umano Onlus APS, 00166 Roma, RM, Italy; 3 Occupational Health, Local Health Authority ASL Roma 4, 00053 Civitavecchia, RM, Italy; martina.gasbarri@aslroma4.it; 4 Workplace Safety Prevention Service, Marche Regional Health Authority, AREA VASTA 3, 62100 Macerata, MC, Italy; lucia.isolani@sanita.marche.it

**Keywords:** health promotion, total worker health, metabolic syndrome, hypertension, dyslipidemia, hyperglycemia, obesity, depression, happiness, sleep disorders

## Abstract

**Background/Objectives**: The Mediterranean diet (MD) is a set of customs and eating habits developed in countries bordering the Mediterranean Sea thousands of years ago. These habits are frequently proposed as a complement to pharmacological therapies for chronic diseases and could also counteract their onset. This study aimed to investigate adherence to the MD in workers from 22 companies in Latium (Italy) and to evaluate the association of diet with workers’ health. **Methods**: Workers undergoing their health surveillance examination and routine blood tests (total cholesterol, HDL cholesterol, triglycerides, glucose) were invited to fill in the Mediterranean Diet Adherence Screener (MEDAS), the Effort–Reward Imbalance (ERI) questionnaire for work-related stress, the Sleep Condition Indicator (SCI) for sleep quality, the STOP-Bang questionnaire for obstructive sleep apnea (OSA) risk, the General Health Questionnaire (GHQ12) for low mental health, and the Abdel-Khalek questionnaire (AKQ) for happiness. A total of 1475 out of 1608 workers (91.7%) participated in the study. **Results**: The prevalence of high adherence to the MD was 53.8% (95% CI 51.3; 56.4). Female workers reported greater adherence to the MD than males (Mann–Whitney U, *p* < 0.001). MD adherence was positively correlated with age (Spearman’s rho = 0.071, *p* < 0.01). Workers who had never smoked and those who engaged in physical activity during their leisure time showed significantly higher adherence (*p* < 0.001). In multiple logistic regression models adjusted for age, gender, smoking status, physical activity, night work, and occupational sector, the MEDAS score was inversely associated with hypertension [aOR = 0.922 (95% CI = 0.856; 0.994)], hypertriglyceridemia [aOR = 0.919 (95% CI = 0.844; 0.999)], and metabolic syndrome [aOR = 0.911 (95% CI = 0.831; 0.999)]. The MEDAS score was positively related to happiness [aOR = 1.074 (95% CI = 1.017; 1.134)]. **Conclusions**: Although the association between diet adherence and health is weak, the MD remains a useful measure for promoting health in the workplace.

## 1. Introduction

Workplace health surveillance provides an opportunity for bringing many people together in a setting with medical facilities and demonstrates the advantage of combining legally mandated medical checkups, aimed at preventing occupational risks, with health promotion programs. This approach, which is implemented in Italy as Promotion included in Prevention (PIP) [1] and in the US as Total Worker Health© (TWH) [2], is characterized by principles of autonomy (the worker can refuse to participate), universality (all workers are eligible), and free access for all workers. Most of these programs are aimed at identifying and preventing chronic diseases [3,4,5]. The effectiveness of these types of interventions in the workplace setting, however, is sparse and inconsistent [6]. To increase effectiveness and sustainability, some researchers suggest encouraging worker participation [7,8] and focusing on positive outcomes, such as wellbeing, quality of life or satisfaction [9,10,11].

For over 20 years, we have followed these principles by implementing health participatory promotion initiatives in the workplace, even in small and very small companies, preferably with a salutogenic approach, and have consistently achieved nearly 100% employee participation [12]. Our 2022 nutritional health project revealed that many workers had poor eating habits and a poor knowledge of the principles of proper nutrition [13]. Health eating awareness has emerged as a crucial factor against disordered eating [14]. Research has demonstrated the effectiveness of MD promotion programs in enhancing community adherence and reducing metabolic risk factors [15,16,17,18]. From the standpoint of the health system, the MD is an affordable intervention that could result in significant long-term savings [19]. For all these reasons, in 2023, in consultation with the workers, we planned a program to promote the MD in all workplaces under our medical supervision.

The term “Mediterranean diet” encompasses a series of dietary practices that originated thousands of years ago in the Mediterranean basin and that continue to be largely followed by the people living on its shores. The MD consists mainly of plant-based foods (wheat, fruits, vegetables, olive oil), a low consumption of animal products, and a moderate consumption of wine during meals. There are regional differences in the way local products are grown, cooked, and eaten. The properties of the MD were discovered after the Second World War and given prominence by the Seven Countries Study [20]. In 2010, the diet was included on UNESCO’s Representative List of Intangible Cultural Heritage of Humanity [21].

Its positive effects may be attributed to several physiological processes, such as increased endothelial function, antithrombotic activity, reduced oxidative stress and inflammatory indicators, improved insulin sensitivity, and favorable modification of lipid profiles. Bioactive substances like polyphenols, dietary fiber, and monounsaturated and polyunsaturated fatty acids are primarily responsible for these benefits [22], acting on the gut microbiome and plasma metabolome [23,24,25]. Systematic reviews indicate that MD improves HbA1c [26], LDL cholesterol, and triglyceride levels [27], homeostatic model assessment of insulin resistance (HOMA-IR) index, and total, low-density lipoprotein (LDL), and high-density lipoprotein (HDL)cholesterol [28], and may be a favorable strategy for targeting hepatic steatosis and lipid metabolism disorders [29]. High adherence to the MD is associated with reduced mortality in people with pre-existing metabolic illnesses as well as moderate but clinically significant improvements in metabolic health [30]. In comparison with other diets, it generally achieves better results [31,32]. It may also reduce body mass index, waist circumference, triglycerides, and the fatty liver index score in overweight or obese subjects more efficiently than alternative diets or recommendations [33].

Its anti-inflammatory potential may help prevent and slow down the evolution of cardiovascular diseases [34,35] by lowering serum high-sensitivity *C*-reactive protein (hs-CRP) levels and by favorably influencing the reduction in cardiovascular (CVD) risk factors such as blood pressure and lipid profiles [36]. Scientific evidence supports the use of the Mediterranean Dietary Approaches to Stop Hypertension (DASH) diet [37]. The DASH diet is a workable strategy for managing blood pressure, hypercholesterolemia, and weight loss [38]. More generally, the MD produced beneficial effects not only on all components and most risk factors of metabolic syndrome (MetS), but also on CVD and stroke incidence [28].

The antioxidant effect of the MD also has beneficial effects on the production of melatonin and consequently on sleep [39]. Compared to people with insufficient sleep duration, poor-quality sleep, and later chronotype, individuals with sufficient sleep duration, good-quality sleep, and earlier chronotype had considerably greater probabilities of having high MD scores [40]. Moreover, the MD has a reciprocal relationship with sleep disorders such as obstructive sleep apnea (OSA) and insomnia. A prospective study showed that there is significant interaction between the MD and sleep disorders, and this influences cardiovascular mortality [41]. The ATTICA cohort study showed that the protective association between MD adherence and cardiovascular risk was found only in participants who slept adequately [42]. According to a growing body of scientific research, increasing adherence to the MD is linked to positive results for mental health [43,44,45,46]. A review of 126 studies with over 8 million participants found modest evidence that high adherence to the MD, as well as a one-point increase in the adherence level, are significantly associated with a small reduction in the risk of several site-specific cancers and of cancer-related mortality [47]. A meta-analysis of 54 longitudinal studies indicated that a one-point increase in adherence corresponds to a relative risk (RR) of all-cause mortality of 0.96 (95% CI: 0.95–0.97), indicating a significant protective effect [48].

Aging of the workforce, which goes hand in hand with that of the world population, means that chronic pathologies are increasingly common among working people, especially those with low-paid jobs. Poor adherence to the MD is associated with low economic status and poor quality of life [49]. Poor eating habits have been reported in workers from a variety of economic sectors worldwide [50,51,52]. This has led to absenteeism and lost productivity with consequent high costs for employers and companies [53,54]. Poor eating habits have been linked to a variety of occupational situations such as stress at work, long hours, working shifts, and a lack of ingredients that support a healthy diet [55,56]. Promoting a healthy diet in the workplace is a classic win–win situation: workers improve their health, companies increase productivity, and health services fulfill their institutional mandate to foster good practices and worker participation. Advocating healthy dietary practices is part of many workplace health promotion programs, even if there is limited evidence on effectiveness [3,6].

For these reasons, we deemed it appropriate to investigate the degree of adherence to the Mediterranean diet among the workers of the companies we monitored. We used the MEDAS (Mediterranean Diet Adherence Screener) questionnaire, a validated tool widely used in numerous countries [57]. The primary aim of the research was therefore to evaluate adherence to the MD in the Latium region and to verify whether there was any difference based on gender, age, or work sector. A second objective was to assess the association between the degree of adherence to the diet and the main metabolic and mental health parameters used in occupational medicine.

The hypotheses we formulated before the MD promotion campaign were:

1. MD adherence is inversely associated with MetS and its components (obesity, hypertension, hyperglycemia, hypercholesterolemia, hypertriglyceridemia);

2. MD adherence is inversely associated with the risk of OSA and insomnia;

3. MD adherence is inversely associated with the risk of distress, anxiety, depression, and unhappiness.

## 2. Materials and Methods

### 2.1. Population

A health promotion program aimed at encouraging adherence to the MD was extended to all the workers exposed to occupational risk factors who were called in for periodic medical checkups intended to prevent risks in 2023. Information on their eating habits was collected using the tools described in this article. After collecting the questionnaires, they were given training on the principles of proper nutrition by means of seminars and the distribution of leaflets.

At the end of their annual medical examination, out of the 1608 workers undergoing health surveillance, 1475 responded to the invitation to take part in the health promotion program (91.7% participation rate). The workers were employed in different companies, including the commercial (111, 7.5%), industrial (49, 3.3%), social (260, 17.6%), and health (1055, 71.5%) sectors. There were more female employees (1009, 68.4%) than male employees (466, 31.6%). The average age was 47.21 ± 11.20.

The project was approved by the University Department of Life Sciences and Public Health on 20 January 2023 (PROT. No. APROV. IST DIPUSVSP-22-02-239) and by the Ethics Committee of the Università Cattolica del Sacro Cuore, Policlinico A. Gemelli, Rome, on 23 February 2023 (ID 5570 prot.n.0006903/23).

### 2.2. Questionnaire

To evaluate adherence to the MD, we used the Italian version [57] of the 14-point Mediterranean Diet Adherence Screener (MEDAS) [58]. This questionnaire, containing 14 dichotomy questions, was developed within the Prevención con Dieta Mediterránea (PREDIMED) study group to conveniently determine adherence to the traditional MD and provide patients with immediate feedback [59]. The score, which expresses the degree of adherence to the MD, ranges between 0 and 14. A score equal to or above 9 indicates high adherence [60].

The Italian version [61] of Siegrist’s Effort–Reward Imbalance (ERI) questionnaire [62] was used to investigate work-related stress. The shortened form of the questionnaire was adopted for this investigation [63]. The Italian version consists of a total of ten questions, each requiring a response on a 4-point Likert scale. With a score ranging from 3 to 12, three questions gauge the amount of effort employees put into their work, while the remaining seven questions gauge the tangible or intangible benefits that they receive from their jobs (scores ranging from 7 to 28). The weighted ratio of effort to reward (ERI) is used to calculate stress. Distress is indicated by a score greater than 1. In this study, Cronbach’s alpha for effort and reward was 0.841 and 0.746, respectively.

Sleep quality was assessed using the Italian version [64] of the Sleep Condition Indicator (SCI) [65], short version (SCI-2) [66]. This consists of two questions; responses on a 5-point Likert scale result in a score ranging from 0 to 8. High scores indicate better sleep quality, while scores below 4 indicate a risk of insomnia. In this study, the reliability of the questionnaire, measured as Cronbach’s alpha, was 0.835.

We used two binary questions about snoring and observed apnea from the STOP-Bang questionnaire [67,68] to assess the risk of suffering from obstructive sleep apnea (OSA) according to recommendations for large surveys of the Italian Interdisciplinary Technical Committee for Sleepiness and Safety in OSAS Patients [69].

The GHQ12, an abbreviated version of the General Health Questionnaire, was used to detect mild mental health issues such as anxiety and depression symptoms, or other symptoms that require a more clinical evaluation in order to provide early detection of possible psychiatric disorders [70,71,72]. The questionnaire has twelve 4-point items; responses can be rated using a Likert scale (1-2-3-4) or a binary scale (0-0-1-1) [73]. We chose the Likert scale for which the cut-off is 11 points [74,75]. In this study, Cronbach’s alpha for the questionnaire was 0.878.

Happiness level was measured according to the Abdel-Khalek questionnaire (AKQ) [76] using a single item rated from 0 to 10. Since this one-item questionnaire lacks a cut-off value, we dichotomized the scores at the median.

During periodic medical examinations, the measurements of anthropometric data (height, weight, and waist circumference) followed criteria established by the International Society for the Advancement of Kinanthropometry (ISAK) [77]. The body mass index (BMI) was calculated by dividing a worker’s weight in kilograms by her/his height in square meters [78]. According to the 2023 ESH European Hypertension guideline update [79,80], hypertension was defined as systolic pressure ≥ 140 mmHg, diastolic pressure ≥ 90 mmHg, or continuous antihypertensive treatment.

Triglycerides, total cholesterol, HDL cholesterol, and blood glucose were measured. The International Diabetes Federation (IDF) [81], the National Cholesterol Education Program Expert Panel on the Detection, Evaluation, and Treatment of High Cholesterol in Adults (NCEP/ATPIII) [82], the American Association of Clinical Endocrinologists (AACE) [83], and the Joint Societies Guidelines on Management of Cholesterol [84] were taken into consideration when defining cut-off levels of metabolic parameters. Hypercholesterolemia was defined as total cholesterol exceeding 200 mg/dL (5.2 mmol/L), HDL cholesterol below 40 mg/dL (1.03 mmol/L) in men and below 50 mg/dL in women, or treatment for hyperlipidemia. Hypertriglyceridemia was defined as a serum triglyceride level of over 150 mg/dL (1.7 mmol/L). High fasting glucose was defined as a plasma glucose level greater than 100 mg/dL (5.6 mmol/L) or the use of hypoglycemic medication. Metabolic syndrome (MetS) was defined according to the National Cholesterol Education Program Adult Treatment Panel III (NCEP ATP III) criteria [82], requiring the presence of any 3 or more of the above reported 5 risk factors: abdominal obesity, elevated triglycerides, reduced HDL cholesterol, elevated blood pressure, and elevated fasting glucose.

No generative artificial intelligence (GenAI) has been used in this paper to create text, data, or graphics, or to assist in study design, data collection, analysis, or interpretation. The dataset used in this study has been deposited in a publicly available database (Zenodo, accession date 30 August 2026, https://doi.org/10.5281/zenodo.22176706).

### 2.3. Data Analysis

The Anderson–Darling test [85] was used to verify whether the variables of interest followed a normal distribution. Nonparametric methods were used for variables that did not follow a normal distribution; however, as suggested by Lumley [86], the sample size convinced us that parametric methods could be used even when this assumption was not met. Consequently, to compare the MEDAS score values in the different categories, we used the Mann–Whitney U test for two groups or the Kruskal–Wallis test for multiple groups, but similar results were obtained with the Student’s *t*-test or with the ANOVA analysis of variance and the Bonferroni post hoc test. The effect size of differences was assessed with Cohen’s D.

To assess the prevalence of pathological conditions, continuous variables were dichotomized using the questionnaire cut-off, the median value, or the normal limits of blood tests. Fifty years was taken as the age limit for the elderly. The Clopper–Pearson exact test was used to establish 95% confidence intervals (95% CI) of the prevalences, and the chi-square test was adopted to compare proportions.

Initially, we examined the distribution of high versus low dietary adherence across various population subgroups (age, gender, smoking status, physical activity, night work, and occupational sector), and compared MEDAS scores within these subgroups, also assessing the effect sizes of the comparisons. We then constructed multivariate models to evaluate the association between MEDAS scores and various health problems. To assess the association between the degree of adherence to the MD and metabolic and psychosocial variables, we constructed multiple logistic regression models with the presence/absence of pathology as the dependent variable and the MEDAS score, age, gender, night shift, smoke, physical activity, and work sector as independent variables, thus obtaining the adjusted odds ratio (aOR) and 95% confidence intervals (95% CI).

Statistical analyses were performed using version 31.0 of the IBM Corp. (Armonk, NY, USA) (2026). IBM SPSS Statistics for Windows (Version 31.0), supplemented by R’s MVN package, version 6.3.

## 3. Results

### 3.1. MEDAS Score Distribution

The MEDAS questionnaire provided values ranging from 2 to 14, with a median value of 9 points, which corresponds to the cut-off for high adherence to the MD. The Anderson–Darling test indicated a significant deviation from normality (A^2^ = 14.558, *p* < 0.001) (Figure 1). The distribution had negative skewness (=−0.114), with extreme values lower than the mean. The distribution curve was platykurtic (kurtosis = −0.237). The mean score of the MEDAS questionnaire was 8.66 ± 2.05.

In the sample, 793 workers achieved or exceeded nine correct answers on the MEDAS questionnaire, thereby indicating high adherence to the MD. The prevalence of high adherence was 53.8 (95% CI 51.3; 56.4).

The questions that showed the greatest non-adherence to MD principles concerned the consumption of fish and shellfish (only 32.6% reported consuming the latter three or more times a week) and also butter, margarine or cream, since only 29.7% avoided consuming more than a teaspoon a day, even though olive oil was the main condiment for 95.8%. In fact, many respondents (34.6%) reported olive oil consumption lower than the four tablespoons per day indicated by MD standards. Also, less than the recommended quantity of fruit and legumes was consumed by 36.7% and 46.0% of participants, respectively (Table S1).

Female workers reported greater adherence to the MD than males, scoring 8.82 ± 2.01 and 8.32 ± 2.11, respectively (Mann–Whitney U, *p* < 0.001; Student t, *p* < 0.001). The effect size of the difference was small when measured using Cohen’s D (D = −0.245; CI95% −0.355; −0.134). There were 568 high adherents to the MD (56.3%) among women, while male adherents were 225 (48.4%) (Pearson’s chi square *p* = 0.004).

MD adherence was positively correlated with age (Spearman’s rho = 0.07 *p* < 0.01). Younger workers, aged up to 50 years, had a lower prevalence of high adherence to the MD compared to those over 50 years of age (chi square *p* < 0.05). The comparison of mean MEDAS scores between the two groups—those above and below 50 years of age—was marginally significant (8.59 vs. 8.76, *p* = 0.05), but the effect was very small (Cohen’s D = −0.085, CI95% −0.188; −0.017). No significant difference in the MEDAS score was observed between workers who performed night work (n = 310) and the others (n = 1163). The percentages of low and high adherence to the MD were comparable between those who did and did not perform night work (chi square *p* = 0.298). Mean MEDAS scores in these two groups were also similar; night shift workers reported slightly higher adherence than the others, but the difference was not significant (Student’s *t*-test 8.72 ± 2.12 vs. 8.65 ± 2.03, *p* = 0.593).

Smoking habits appeared to be associated with dietary habits. Those who had never smoked had a significantly higher rate of high adherence to the Mediterranean diet than former and current smokers (chi square *p* < 0.001). The mean MEDAS score of those who had never smoked was significantly higher than that of the others (8.91 ± 2.04 vs. 8.46 ± 2.04, Student’s *p* < 0.001; Mann–Whitney U *p* < 0.001). The effect size was small (Cohen’s D = 0.219, CI95% 0.116; 0.322).

The frequency of physical exercise in leisure time was correlated with adherence to the MD (Spearman’s rho = 0.144; *p* < 0.001). Inactive workers (n = 546) had a higher prevalence of low adherence to the Mediterranean diet compared to those who habitually engaged in vigorous physical activity at least once a week (chi square *p* < 0.001). The mean MEDAS score among inactive workers was significantly lower than that of those who engaged in regular physical activity (Mann–Whitney U *p* < 0.001; Student’s t *p* < 0.001), with a low/medium effect size (Cohen’s D = 0.300; CI95% −0.406; −0.193).

A comparison of the employment sectors revealed significant differences in the degree of adherence to the MD (one-way ANOVA *p* = 0.004). On average, industrial workers had a significantly lower level of adherence (7.71 ± 2.14) than social (8.67 ± 2.12, *p* < 0.002) and healthcare workers (8.73 ± 2.02, *p* < 0.001). In the social and healthcare sectors, the majority of workers reported high adherence to the MD, while the opposite occurred in retail and industrial workers, who showed the lowest adherence to the principles of the diet (Table 1).

Given that many workers (452, 30.6%) were already being treated for one or more metabolic disease and had presumably been advised by their doctors to follow a proper diet, we examined whether there was a difference in dietary adherence between this group and the other workers. The workers undergoing treatment for metabolic diseases had a mean MEDAS score of 8.75 ± 2.04, not significantly different from that of the other workers (8.62 ± 2.06, *p* = 0.272).

### 3.2. Association of Diet with Metabolic Diseases

Laboratory tests and physical examinations indicated that 332 workers (22.5; 95% CI 20.4; 24.7) had abdominal or central obesity. Furthermore, 274 (prevalence 18.6, 95% CI 16.7; 20.7) had high blood pressure or were undergoing treatment for hypertension. Dyslipidemia (low HDL cholesterol, elevated total cholesterol, or ongoing treatment) was reported by 351 participants (23.9; 95% CI 21.7; 26.2), and hypertriglyceridemia by 170 workers (11.6; 95% CI 10.0; 13.3). A total of 161 workers (10.9; 95% CI 9.5; 12.8) had hyperglycemia or were undergoing treatment for it. Workers who were already undergoing treatment at the time of observation were: 184 (12.5%) for blood pressure, 181 (12.3%) for cholesterol, 53 (3.6%) for triglycerides, 66 (4.5%) for glucose, and 245 (16.6%) for weight. Approximately half of the sample (729, 49.4%) presented with at least one metabolic disorder, and 145 individuals (9.9; 95% CI 8.4; 11.5) were suffering from at least three of these conditions at the same time (MetS patients). Overall, 452 workers (30.6% of the total) were being treated for one or more metabolic disorders.

We examined the association between the degree of adherence to the MD and the presence of each metabolic disease using Pearson’s chi square (Table 2) and Mann–Whitney U or Student’s *t*-test. Workers without metabolic disorders had a higher degree of adherence to the diet; however, differences failed to reach statistical significance and merely approached it for waist obesity and hypertriglyceridemia. Hypertensive individuals had, on average, a lower MEDAS score than their counterparts (8.49 ± 2.08 vs. 8.70 ± 2.04), but the difference was not significant (*p* = 0.121). Similarly, adherence to the MD among workers with low HDL cholesterol was lower on average than among the others (8.56 ± 2.04 vs. 8.69 ± 2.05), but the difference was not significant (*p* = 0.311). Workers with hyperglycemia also reported lower average scores than the others (8.45 ± 2.13 vs. 8.68 ± 2.04), though the difference was not significant (*p* = 0.191). A comparison of MEDAS scores in workers with hypertriglyceridemia and those with normal triglycerides using the Mann–Whitney U test indicated that the null hypothesis of equivalence should be rejected (*p* < 0.007). Workers with normal triglycerides reported a mean MEDAS score of 8.71 ± 2.03, while those with high triglycerides scored 8.24 ± 2.1; the difference was highly significant also using a two-tailed Student *t*-test for unassumed equal variances (*p* < 0.007). The effect size was small (Cohen’s D = 0.227; CI95% 0.067; 0.388). Workers with abdominal obesity reported significantly lower adherence to the diet than their colleagues (8.46 ± 2.13 vs. 8.72 ± 2.03; *p* = 0.044), with a small effect size (Cohen’s D = 0.126; CI95% 0.004; 0.248). Finally, workers with metabolic syndrome reported significantly lower adherence than other workers (8.33 ± 2.14 vs. 8.69 ± 2.03; *p* = 0.045), with a small effect size (Cohen’s D = 0.176; CI95% 0.004; 0.347).

Using multiple logistic regression models, we assessed the association between diet and metabolic diseases. The MEDAS score was significantly associated with hypertension [aOR = 0.9223 (0.856; 0.994)], hypertriglyceridemia [aOR = 0.919 (0.844; 0.999)], and metabolic syndrome [aOR = 0.911 (0.831; 0.999)]; the relationship approached significance in the case of hypercholesterolemia, hyperglycemia and obesity (Table 3).

### 3.3. Association of Diet with Psychosocial Variables

We studied the association between diet and variables by measuring work-related stress, insomnia, psychological distress and happiness. Questions related to these variables had a slightly higher rate of incomplete responses than the others. For example, 1389 people answered all work-related stress questions, while 86 respondents (5.8%) provided incomplete or missing answers. Since incomplete responses to other sections of the questionnaire were rare, we interpreted this as reluctance to provide information on perceived stress. Among the respondents, 540 obtained an ERI score higher than one, thereby indicating distress, or an imbalance between effort and rewards. We can therefore affirm that 38.9% of workers (95% CI 36.3; 41.5) perceive a discrepancy between the efforts made and the rewards they receive for the work done.

Symptoms of insomnia were reported by 332 workers (22.8%; 95% CI 20.6; 25.0). Workers with good sleep quality had a MEDAS score similar to those with symptoms of insomnia or poor sleep quality (8.67 ± 2.09 vs. 8.63 ± 1.67) (Mann–Whitney U *p* = 0.763).

Suspected OSA was observed in 193 workers (13.1; 95% CI 11.4; 15.0). These workers had a slightly lower MEDAS score than those who had no symptoms (8.46 ± 1.92 vs. 8.70 ± 2.07); however, the difference was not significant (Mann–Whitney U = 0.061).

Symptoms consistent with anxiety, depression or other common psychological disorders (GHQ12 score of 12 or more) were reported by 481 people, 32.7% (95% CI 30.3; 35.2). Workers in good mental health reported a higher mean MEDAS score than those who were at risk for mental health problems (8.7 ± 2.1 vs. 8.6 ± 1.9); however, the distribution of the MEDAS score in the two groups was not significantly different (Mann–Whitney U *p* = 0.159).

A median value of 7 and a mean of 6.85 ± 1.9 were found for happiness. This variable deviated substantially from normality (Anderson–Darling test = 34.5, *p* < 0.001). It showed a negative asymmetry (−0.895) as demonstrated by the mean, which was lower than the median value, and was leptokurtic (kurtosis = 1.23), with a high possibility of observing extreme values. By dividing the distribution of happiness scores at the median, the MEDAS score distribution in the two groups was significantly different (Mann–Whitney U *p* < 0.003). Workers with happiness levels below the median had a lower average score than the others (8.55 ± 1.99 vs. 8.86 ± 2.13, *p* = 0.004) with a small effect size (Cohen’s D = −0.153; CI95% −0.258; −0.048).

We examined the relationship between the degree of MD adherence and psychosocial variables by using psychosocial conditions as dependent variables and the MEDAS score, gender, age, night shift, smoke, physical activity, and work sector as independent variables. Examination of adjusted odds ratios indicated a significant association with happiness [aOR = 1.074 (95% CI = 1.017; 1.134) *p* = 0.010], (Table 4).

## 4. Discussion

This study, conducted in many different work environments where workers often eat their meals in various locations and lack full control over their diet, substantially confirmed that workers’ adherence to the MD was weakly inversely associated with metabolic and psychological disorders. Cross-sectional observation of dietary habits does not allow for definitive assessments but may provide a useful basis for subsequent promotional interventions. Findings showed that, in complex and diverse settings such as the workplace, adherence to the MD did not result in immediately perceptible health differences. Low risk levels cannot be achieved through diet alone but require an overall improvement in lifestyle. The calculation of odds ratios, adjusted for the effects of age, gender, and other lifestyle factors, showed values close to 0.9, suggesting a modest but favorable association. In no instance was adherence to the MD associated with poorer health status. Although this result cannot be interpreted as establishing a definitive causal link, it, combined with the existing literature, encouraged us to continue promoting the MD among workers.

Our convenience sample, composed of workers from 22 companies in Latium, Italy, reported strong adherence to the MD, with a 53.8% prevalence of high adherence (95% CI 51.3; 56.4), and a 70.9% prevalence of medium-to-high adherence (MEDAS > 8). Previous studies had generally observed lower levels of adherence to the MD, reporting 34% high adherence among schoolteachers in Portugal [87]; 36.2% in over 100,000 Spanish workers undergoing routine health examination [88]; 46.7% in young Turkish adults [89]; 36.4% moderate-to-high adherence in Spanish students [90], and 46.4% moderate-to-high adherence in Spanish adults [91]. We found levels of adherence higher than these only in a research study conducted on a multinational financial company located in Madrid that reported an 89% medium or high MEDAS adherence [92]. It is not surprising that the mean MEDAS score in this sample of workers (8.66 ± 2.05) was much higher than those observed in clinical series, e.g., in polyendocrine metabolic ovarian syndrome (PMOS) (6.8 ± 2.3) [93], female infertility (7.6 ± 1.2) [94], or cancer prevention programs (4.00 ± 1.71) [95]. The MEDAS values observed in our study were within the range of variation that was observed between baseline (8.72 ± 1.82) and follow-up (8.38 ± 1.45) measurement in a population-based longitudinal study conducted in Lombardy, Northern Italy [96].

In our sample, MEDAS scores varied significantly across work sectors. Healthcare and social service workers reported significantly higher compliance than industrial workers. The most likely hypothesis is that this difference was the consequence of a higher level of food and health awareness among healthcare and social service workers compared to industrial workers. Indeed, it is known that literacy, and especially electronic access to information, are associated with the pursuit of healthy lifestyles and greater adherence to the MD [97,98,99,100,101].

The association between MEDAS and age that we observed is intriguing, as aging and cardiovascular risk may overlap in numerous ways, with deregulated nutrient sensing as a common mechanism and lifestyle as a communal modifier [102]. The relationship between age and MEDAS could be attributed to the gradual transition from the MD to continental eating styles that is currently occurring in Mediterranean countries [103] where young people are less inclined to follow the MD than their older colleagues. Studies observing age variation have also suggested another reason—older people possess more cooking skills than young people; in fact, there is a correlation between cooking skills and adherence to the MD [104,105]. In our study, we observed that female workers had a higher MEDAS score than male workers. This could also be due to women having greater cooking skills than men.

In the workers we monitored, MD adherence was inversely associated with MetS and some of its components. The cross-sectional nature of our study does not allow for a definitive interpretation of this association. On the basis of clinical studies reporting the beneficial effects of the MD on anthropometric parameters and metabolic indicators in association with the treatment of chronic diseases, the strength of the association between the MEDAS score and metabolic disorders was lower than one might have expected. However, these studies on patients were not linked to workplaces where it would be useful to understand whether adherence to the MD can prevent the onset of diseases. The weak association we observed does not invalidate the hypothesis that the MD protects against the onset of metabolic diseases. Moreover, although our sample was predominantly composed of healthy workers, we must not overlook the fact that a significant number were chronically treated for metabolic diseases and had probably been given medical advice to follow a specific dietary regimen. Thus, many high adherents to the MEDAS were patients with metabolic diseases who were undergoing treatment related to diet, and this minimized the strength of the association between MEDAS and metabolic wellbeing. Many other workers were likely aware of having altered metabolic markers and were already following advice to watch their diet. Therefore, reverse causality between diseases and diet cannot be ruled out. In conclusion, our study endorses the findings of Rees’ systematic review which concluded that there is little or limited evidence of the moderate benefits of the MD on metabolic and cardiovascular risk factors in primary prevention, and low evidence for secondary prevention [106].

In the complex relationship between nutrition, metabolism, and mental health, sleep definitely plays a critical role. For this reason, we investigated three topics: night shift work, sleep apnea (OSA), and insomnia.

The literature indicates that night workers have an increased metabolic and vascular risk and that the MD may contribute to a risk reduction strategy [107]. However, occupational studies have failed to provide clear indications. One study reported that shift workers consumed more irregular and frequent meals than day workers and often ate at night [108]. In another study, workers with a high frequency of night shifts had a higher energy intake than day workers but followed the same dietary pattern [109]. Conflicting results were also found in Italian studies. Some observed that night workers had higher MEDAS scores than their colleagues [110], while others obtained the opposite result [111]. Studies on shift workers are difficult to compare because numerous parameters, such as the level of activity during night work, the number of shifts within a given timeframe, shift rotation patterns, rest periods, and overtime or double shifts, are inadequately addressed. The self-selection of night workers and the underlying healthy worker effect can be a significant confounding factor. In our study, workers performing night shifts reported slightly higher adherence to the MD than their colleagues, but the difference was not significant. Overall, we deem studies on the MD and night shift work to be inconclusive.

In patients affected by OSA, higher adherence to the MD was associated with a lower likelihood of having insomnia [112]. Combining the MD and weight reduction with traditional OSA treatment has yielded positive results [113]. On the grounds of these findings, the MD is recommended in OSA patients alongside basic therapy, producing some improvement in more severe cases [114]. We have no prospective or retrospective studies that can indicate whether the MD protects against the development of OSA. In our sample, only two workers had been diagnosed with OSA and treated with CPAP, while the others had not been diagnosed or treated, although they manifested symptoms of apnea. However, apneic workers reported lower adherence to the MD than colleagues, but the difference was not significant.

In the literature, higher adherence to the MD is associated with better sleep outcomes [115,116]. Studies performed outside the Mediterranean basin revealed a connection between sleep quality and adopting a Mediterranean-style diet, indicating the possibility of biological processes supporting this relationship [117]. However, the causal relationship between the MD and good sleep has not yet been proven [118]. Most of the research did not examine the directionality or causal links between diet and variations in sleep quality [116]. Moreover, well-conducted studies have found no association between diet and sleep duration or quality [119]. In our sample, we found no significant association between insomnia or sleep quality and adherence to the MD.

Psychosocial factors are certainly the most difficult to investigate since objective measures are lacking and the researcher can only evaluate workers’ perceptions and responses. The MD has been linked in various geographical regions to positive mental health effects in adults, such as diminished symptoms of depression and anxiety, decreased perceived stress, and enhanced quality of life and overall wellbeing, applicable to both healthy individuals and those with comorbid conditions. The mechanisms involved include the antioxidant and anti-inflammatory properties of the MD and its influence on gut flora [46]. Nevertheless, the overall quality and reliability of the findings remain constrained by wide variation in assessments, small samples, and significant methodological limitations [120]. We did not find a significant association between the MD and occupational stress but could confirm that MD adherence was positively associated with happiness in life.

In this study, workers with medical conditions consistently reported lower mean dietary adherence scores than others, although the differences were often marginal, with a minimal effect size. Incorporating numerous health-related factors—such as smoking and physical activity—into the multivariate model further reduced the strength of the association.

In conclusion, the results of the associations between the MD and health, although consistent with the literature, were less significant than expected. This may depend on the fact that the “current” MD is an adaptation of the traditional lifestyle rather than a direct continuation [121]. The changing characteristics of foods, rather than adherence to the principles of the diet, make it increasingly difficult to emulate the eating style of many decades ago. For this reason, in some studies, the antioxidant effects of the MD were lower than anticipated [122]. In the absence of new and stronger evidence, or more effective dietary regimens, we believe that promotion of the MD in workplaces should be continued.

The activity model employed in this research can be easily replicated. Data design and analysis are the responsibility of the occupational physician and incur no costs for the company or the workers. The questionnaire was completed immediately prior to the medical examination, while workers were waiting to be seen. The physician was able to review the responses immediately and provide participants with guidance, accompanied by a leaflet on the Mediterranean diet. Data was initially processed for each company and transmitted in an anonymized, aggregated format to the employer, the prevention and protection service, and the workers’ safety representatives, who meet at least once a year as required by law. Subsequently, workers received a summary of the results via email. Finally, we analyzed the aggregated data from all the companies, which forms the basis of this study. The findings were presented during training sessions at the companies most actively involved. These companies are now conducting a new survey in 2026 to assess whether there have been any improvements in diet adherence rates or in metabolic and mental health parameters.

Our study, in addition to evaluating MD adherence among workers from various companies in Latium, sheds light on the relationship between adherence to diet principles and physical and mental health. Despite the large number of workplace health promotion programs that aim to improve diet, there are few studies on this topic. The data presented in this work is a basis on which to build intervention to improve workers’ health over time. The health surveillance service in companies is strongly interested in promoting health and reducing the productive burden of chronic diseases. Pharmacological treatment is the responsibility of the attending physician, but the occupational physician can support this effort by disseminating useful practices, correct lifestyles and relaxation techniques. Diet and exercise, with a combination of energy reduction and control over the percentage of macronutrient intake, along with regular structured exercise and stress reduction, is the generally practiced route [123]. Effectively reducing risk factors depends largely on patient motivation which improves lifestyle adherence. The occupational physician can provide ongoing support and counseling. Regular interpersonal interaction with workers is the mainstay of all treatment [124].

This study has several strengths. The main one is that it investigated one topic, i.e., adherence to the MD and its relationship with many indicators of physical and mental health in the workplace. To the best of our knowledge, we are not aware of other studies that have simultaneously considered so many factors. Promotional action included in routine prevention is not common, but it provides a way of revealing the health status of the population without resorting to significant financial resources. This study had the advantage of recruiting almost all the workers at a very low cost that did not require funding from companies or public agencies. Programs used for data collection and statistical analysis methods are available free of charge to doctors interested in this kind of research. The method can easily be duplicated by other occupational physicians. Collecting larger samples may help to better clarify the value of the MD in primary and secondary prevention.

However, this advantage was accompanied by several limitations. Firstly, the convenience sample was composed predominantly of healthcare and social service workers, with a female majority; this limited the possibility of referring results to different cultural, social, and economic occupational environments. Obviously, conducting the survey as part of a nutritional health promotion program included in mandatory health surveillance reduced costs and made it possible to verify the effectiveness of MD promotion. These observations could be extended to other production settings if other occupational physicians were willing to apply this method.

Secondly, the specific issues associated with studies conducted in workplaces must be carefully evaluated. While studying an employed population, the possible healthy worker effect must always be considered; the results may differ from those of the general population. The very high participation rate in this, and in other workplace studies, is a strength in terms of participation among eligible workers, but it does not eliminate the selection inherent in studying an actively employed population undergoing occupational health surveillance. Clearly, the voluntary nature of participation does not eliminate the problem of self-selection bias. The possibility that responses are influenced by social desirability must also always be considered. Collecting voluntary information during occupational health surveillance requires conditions that are not always met in every workplace. First, data collection tools must be simple and quick to complete; workers must not be hindered in their productive tasks, and occupational health examinations themselves must fit within schedules dictated by work operations. Workers need to be motivated to participate. Therefore, positive health goals, such as improving one’s diet, are preferable to topics that might trigger anxiety, such as disclosing an illness or addiction that could compromise one’s ability to work or find employment.

Thirdly, this exploratory study investigated the relationship between diet adherence and numerous variables, choosing only a few parameters for each. In some cases, the variable was measured by a single item; this certainly reduced statistical validity. No other options were possible due to the limited time available for health surveillance examinations which have to be conducted without causing excessive disruption to production activities. Further studies could adopt a greater number of indicators and combine instrumental and laboratory measurements to examine metabolic or psychological disorders separately.

Fourthly, as in all cross-sectional studies, it is not possible to determine the direction of the associations. Also in this case, a solution could be found by choosing health surveillance as the study setting. Cohort studies could be performed by repeating health surveillance examinations with the same workers; this is an advantage of workplace studies and would be a logical extension of this investigation.

## 5. Conclusions

Workers from numerous companies in Latium, who undergo mandatory health surveillance, have a good level of adherence to the MD. Adherence was higher among women and older workers but lower among smokers and those who did not engage in sports during their leisure time, and was associated with a slightly lower prevalence of metabolic diseases, and a non-significantly lower level of sleep and mental health problems. Although the association between diet adherence and health is weak, the MD remains a useful measure for promoting health in the workplace.

## Figures and Tables

**Figure 1:**
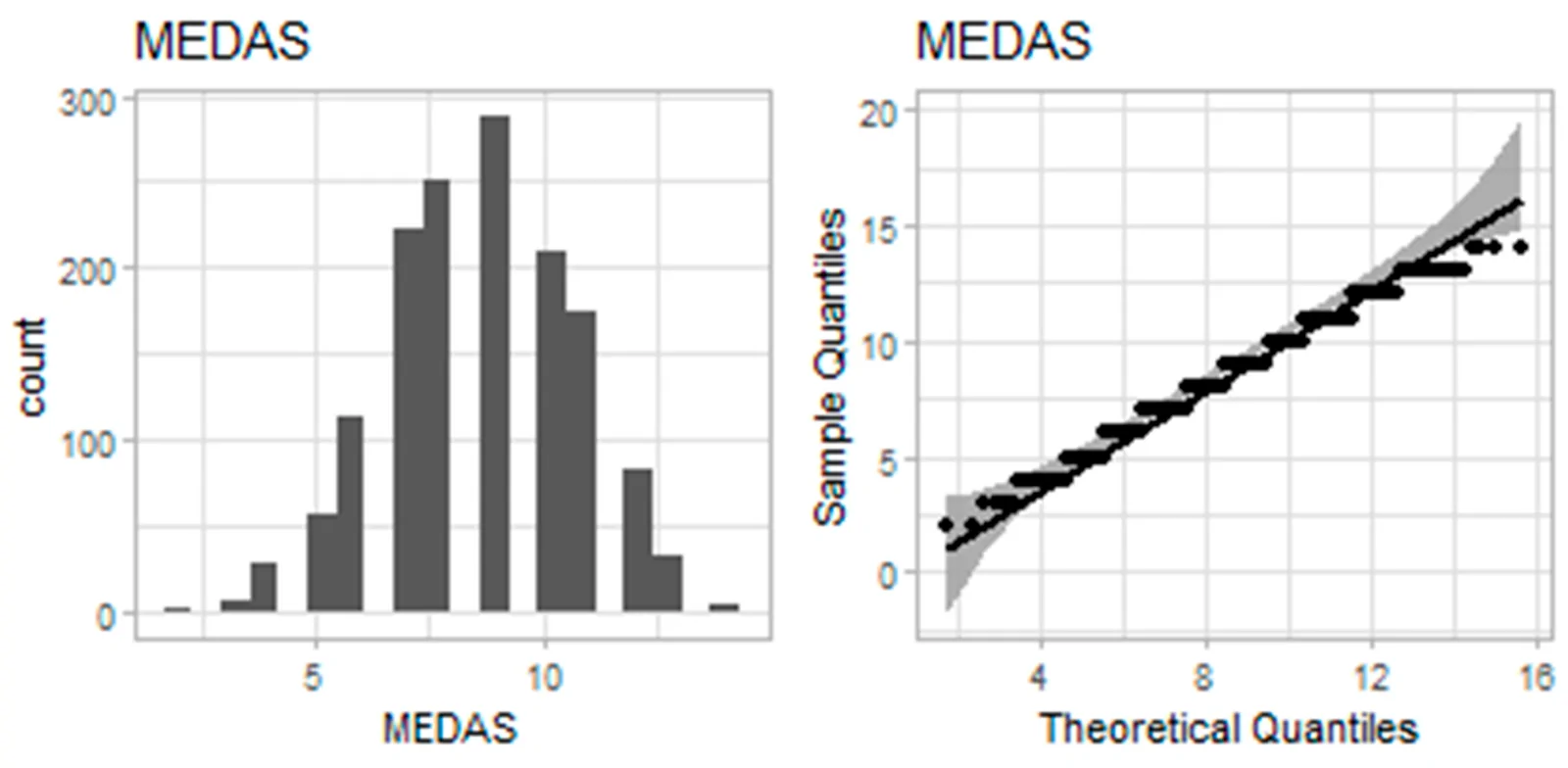
Count of observed scores (**left**) and Q-Q plot (**right**), comparing the quantiles of the MEDAS distribution with the ideal Gaussian distribution.

**Table 1:** Comparison of the frequency of low and high adherence to the MD across genders, age groups, work shift types and occupational sectors.

Variable	Low Adherence to MD N (%)	High Adherence to MD N (%)	*p*
Gender, male	240 (51.6%)	225 (48.4%)	0.004 ^1^
female	440 (43.7%)	568 (56.3%)	
Age: young	397 (48.7%)	419 (51.3%)	0.033 ^1^
Aged (>50 yr)	283 (43.1%)	374 (56.9%)	
Shift: no night work	545 (46.9%)	618 (53.1%)	0.298 ^1^
Night work	135 (43.5%)	175 (56.5%)	
Smoke: never	276 (40.8%)	400 (59.2%)	
Former or current smoker	400 (50.4%)	393 (49.6%)	<0.001 ^1^
Sport in leisure time: never	298 (54.6%)	248 (45.4%)	
1–3 or more times a week	375 (41.3%)	533 (58.7%)	<0.001 ^1^
Sector: retail	56 (50.5%)	55 (49.5%)	0.042 ^1^
Industrial	31 (63.3%)	18 (36.7%)	
Social	125 (48.1%)	135 (51.9%)	
Healthcare	468 (44.4%)	585 (55.6%)	

^1^ chi square.

**Table 2:** Comparison of the prevalence of metabolic disease in workers with low and high adherence to the diet.

Variable	Low Adherence to MD N (%)	High Adherence to MD N (%)	*p*
Elevated blood pressure	132 (19.4%)	142 (17.9%)	0.466 ^1^
Low HDL cholesterol	169 (24.9%)	182 (23.1%)	0.424 ^1^
Hypertriglyceridemia	90 (13.2%)	80 (10.2%)	0.067 ^1^
Hyperglycemia	80 (11.8%)	81 (10.4%)	0.370 ^1^
Obesity	158 (23.2%)	174 (21.9%)	0.554 ^1^
MetS	70 (10.3%)	75 (9.5%)	0.613 ^1^

^1^ chi square.

**Table 3:** Association between the MEDAS score and metabolic disease. Multiple logistic regression models adjusted for age, gender, night shift, smoke, physical activity, and work sector.

Metabolic Disease	aOR ^1^ (95% CI)	*p*
Hypertension	0.922 (0.856; 0.994)	0.033
Cholesterol dyslipidemia	0.948 (0.887; 1.012)	0.108
Hypertriglyceridemia	0.919 (0.844; 0.999)	0.050
Hyperglycemia	0.935 (0.858; 1.020)	0.131
Obesity	0.968 (0.908; 1.031)	0.313
MetS	0.911 (0.831; 0.999)	0.048

^1^ Adjusted for age, gender, night shift, smoke, physical activity, and work sector.

**Table 4:** Association between the MEDAS score and psychosocial variables. Multiple logistic regression models adjusted for age, gender, night shift, smoke, physical activity, and work sector.

Psychosocial Variable	aOR ^1^ (95% CI)	*p*
Work stress (ERI)	1.025 (0.968; 1.087)	0.394
Insomnia (SCI)	1.017 (0.952; 1.085)	0.618
Suspected OSA	0.959 (0.886; 1.037)	0.294
Low mental health (GHQ12)	0.952 (0.898; 1.009)	0.095
Happiness	1.074 (1.017; 1.134)	0.010

^1^ Adjusted for age, gender, night shift, smoke, physical activity, and work sector.

## Data Availability

The dataset used in this study has been deposited in a publicly available database (Zenodo, accession date 30 August 2026, https://doi.org/10.5281/zenodo.22176706).

## Supplementary Materials

The following supporting information can be downloaded at https://www.mdpi.com/article/10.3390/nu18193132/s1, Table S1: MEDAS questionnaire and percentage of adherence to each question.

## Abbreviations

The following abbreviations are used in this manuscript:

95% CI	Confidence intervals at 95%
AACE	American Association of Clinical Endocrinologists
AKQ	Abdel-Khalek questionnaire
aOR	Adjusted odds ratio
BMI	Body mass index
CVD	Cardiovascular disease
DASH	Mediterranean Dietary Approaches to Stop Hypertension
ERI	Effort–Reward Imbalance
GHQ12	General Health Questionnaire-12
HbA1c	Glycated hemoglobin
HDL	High-density lipoprotein
HOMA-IR	Homeostatic Model Assessment of Insulin Resistance
Hs-CRP	High-sensitivity *C*-reactive protein
IDF	International Diabetes Federation
ISAK	International Society for the Advancement of Kinanthropometry
LDL	Low-density lipoprotein
MD	Mediterranean Diet
MEDAS	Mediterranean Diet Adherence Screener
MetS	Metabolic syndrome
miRNA	Micro ribonucleic acid
NCEP/ATPIII	National Cholesterol Education Program Expert Panel on Detection, Evaluation, and Treatment of High Cholesterol in Adults
OR	Odds ratio
OSA	Obstructive sleep apnea
OSAS	Obstructive sleep apnea syndrome
PIP	Promotion in Prevention
PREVIMED	Prevención con Dieta Mediterránea (Prevention with Mediterranean Diet
SCI	Sleep Condition Indicator
TWH	Total Worker Health
