# Supplementary material for: Promoting the Mediterranean Diet in the Workplace: An Observational Cross-Sectional Study
Source: Nutrients. 2026 Sep 23;18(19):3132. doi: 10.3390/nu18193132 (PMC13667134; doi:10.3390/nu18193132)
Supplement: Supplementary file 1 [file nutrients-18-03132-s001.zip › nutrients-4564202-supplementary.pdf]

Table S1. MEDAS questionnaire and percentage of adherence to each question.

N	Answer	Adherence
1	Do you use olive oil as the main source of fat for cooking?	95.8
2	Do you consume at least four tablespoons of extra virgin olive oil per day?	65.4
3	Do you consume at least two servings of vegetables per day? (This includes all cooked and raw vegetables, excluding potatoes and legumes. One serving = a large cup or half a large plate; 1 serving = 200 g - side dishes are 1/2 serving).	66.1
4	Do you consume at least three servings of fresh fruit per day? (One serving = one medium-sized fruit, a large cup of sliced fruit, a medium-sized slice of melon or watermelon, or a cup of freshly squeezed juice).	36.7
5	Do you consume an average of one serving of red meat (pork, veal/beef, lamb, hamburgers, sausages, ham) per day? (One serving = 100-150 g = a quarter to half of a serving plate). (reversed item)	71.9
6	Do you consume, on average, less than one serving of butter, margarine, or cream per day? (One serving = 12 g = one dessert spoonful of butter and margarine; 2 tablespoons for cream).	29.7
7	Do you avoid consuming sugary and/or carbonated beverages? (Sweetened coffee or tea, carbonated drinks with flavors, extracts, or fruit juice, colas, chinotto, lemonade, tonic water, lemonade, orangeade, etc., fruit juice-based drinks, functional drinks with vitamins, mineral salts, and/or energy substances).	75.6
8	Do you drink wine, beer, or alcoholic beverages? (Answer NO if you consume them only exceptionally, <7 glasses per week.)	81.8
9	Do you consume more than three servings of legumes (beans, peas, chickpeas, lentils) per week? (One serving = 150 g = 1 dish.)	46.0
10	Do you consume three or more servings of fish or shellfish per week? (One serving = 100 - 150 g).	32.6
11	Do you eat "commercial" (not homemade) sweets or pastries less than three times a week?	52.8
12	Do you eat more than one serving of unsalted nuts each week? (One serving = 30 g = a handful, including unsalted peanuts, almonds, hazelnuts, chestnuts, walnuts).	53.2
13	Do you usually eat chicken, turkey, rabbit, or vegetarian proteins instead of red meat (veal/beef, pork, lamb) or meat products (hamburgers, sausages, ham)?	85.3
14	Do you eat dishes cooked with tomatoes or tomato sauce, onion and/or garlic, and olive oil more than twice a week?	73.5
